# Comprehensive analysis of different solvent extracts of *Ferula communis* L. fruit reveals phenolic compounds and their biological properties via in vitro and in silico assays

**DOI:** 10.1038/s41598-024-59087-3

**Published:** 2024-04-09

**Authors:** Ghizlane Nouioura, Mohamed El fadili, Azeddin El Barnossi, El Hassania Loukili, Hassan Laaroussi, Mohammed Bouhrim, John P. Giesy, Mourad A. M. Aboul-Soud, Yazeed A. Al-Sheikh, Badiaa Lyoussi, El houssine Derwich

**Affiliations:** 1 Laboratory of Natural Substances, Pharmacology, Environment, Modeling, Health and Quality of Life (SNAMOPEQ), Faculty of Sciences Dhar El Mehraz, Sidi Mohamed Ben Abdellah University, 30 000 Fez, Morocco; 2 LIMAS Laboratory, Faculty of Sciences Dhar El Mehraz, Sidi Mohamed Ben Abdellah University, 30 000 Fez, Morocco; 3 Biological Engineering Laboratory, Faculty of Sciences and Techniques, Sultan Moulay Slimane University, Beni Mellal, Morocco; 4 Laboratory of Biotechnology, Environment, Agri-Food and Health, Faculty of Sciences Dhar El Mahraz, Sidi Mohammed Ben Abdellah University, 30050 Fez, Morocco; 5 Euromed Research Center, Euromed Polytechnic School, Euromed University of Fes, 30 000 Fez, Morocco; 6 Laboratory of Biological Engineering, Team of Functional and Pathological Biology, University Sultan Moulay Slimane, 23000 Beni Mellal, Morocco; 7 Department of Veterinary Biomedical Sciences and Toxicology Centre, University of Saskatchewan, Saskatoon, SK S7N 5B3 Canada; 8 Department of Integrative Biology and Center for Integrative Toxicology, Michigan State University, East Lansing, MI 48895 USA; 9 Department of Environmental Science, Baylor University, Waco, TX 76798 USA; 10 Department of Clinical Laboratory Sciences, College of Applied Medical Sciences, King Saud University, P.O. Box 10219, 11433 Riyadh, Saudi Arabia; 11 Unity of GC/MS and GC, City of Innovation, Sidi Mohamed Ben Abdellah University, Fez, Morocco

**Keywords:** Biochemistry, Biological techniques, Chemical biology, Microbiology

## Abstract

Although giant fennel is recognized as a “superfood” rich in phytochemicals with antioxidant activity, research into the antibacterial properties of its fruits has been relatively limited, compared to studies involving the root and aerial parts of the plant. In this study, seven solvents—acetone, methanol, ethanol, ethyl acetate, chloroform, water, and hexane—were used to extract the chemical constituents of the fruit of giant fennel (*Ferula communis*), a species of flowering plant in the carrot family Apiaceae. Specific attributes of these extracts were investigated using in silico simulations and in vitro bioassays. High-performance liquid chromatography equipped with a diode-array detector (HPLC–DAD) identified 15 compounds in giant fennel extract, with p-coumaric acid, 3-hydroxybenzoic acid, sinapic acid, and syringic acid being dominant. Among the solvents tested, ethanol demonstrated superior antioxidant activity and phenolic and flavonoid contents. *F. communis* extracts showed advanced inhibition of gram-negative pathogens (*Escherichia coli* and *Proteus mirabilis*) and variable antifungal activity against tested strains. Molecular docking simulations assessed the antioxidative, antibacterial, and antifungal properties of *F. communis*, facilitating innovative therapeutic development through predicted compound–protein interactions. In conclusion, the results validate the ethnomedicinal use and potential of *F. communis*. This highlights its significance in natural product research and ethnopharmacology.

## Introduction

The genus *Ferula* belongs to the Apiaceae family of perennial herbaceous plants^1^. It includes approximately 150 species well-suited to diverse Mediterranean and continental climates and grow spontaneously in Morocco^2^. With a documented history of medicinal use, its pharmacological effects are well-established in both human and veterinary practices^3^. Six *Ferula* species have been observed in Morocco: *Ferula communis, Ferula cossoniana, Ferula gouliminensis, Ferula sauvagei, Ferula atlantica, and Ferula tingitana*^4^*.* Known as giant fennel (*Ferula communis* L.) (*la grande Ferule* in French and *l-bubāl* in Arabic) is referred to as *ūffal, tuffalt* in the Berbère language, where its fruit is sold as a vegetable in Moroccan marketplaces (souks)^5^. *F. communis* is renowned for its gum resin (*l-fāsūẖ*)^6^, harvested from the rootstock after stripping and incision^7^. The fruit of *F. communis* is primarily used as a vegetable, prepared by steaming or ashing, and subsequently chopped and seasoned with olive oil, salt, and pepper^8^. *F. communis* grazing is generally avoided by herds, as it can cause poisoning, except during droughts.

This genuine variety is characterized by the synthesis of numerous hydroxycoumarins, including ferulenol^9^. Recent studies highlight the potential of isolated compounds from this genus as chemopreventive agents and in overcoming resistance to anticancer drugs^8,10^. Traditionally, plants of this genus have been used to treat various diseases^9^. Moreover, they exhibit anticonvulsant, stimulant, and expectorant properties^11^. The genus *Ferula* is categorized based on the prevalence of oleo-gum resins (*Asafoetida*) and their roles in natural and conventional medications^12^. *Asafoetida* has been used as a tranquilizer to decrease blood pressure. It is commonly used in Indian food and traditional medicine, particularly within the framework of Indian medical structures such as Ayurveda^8^. *Asafoetida*, a spice with centuries of medicinal use, is employed for therapeutic use. In the Middle Atlas, unexpanded inflorescences of *Ferula* serve as anthelmintics^9^. Polyphenols have well-defined antioxidant properties linked to the inhibition of food oxidation^10^. In addition, these antioxidants effectively block or inhibit the formation and propagation of free radicals^13^. Therefore, they are of interest in several fields, including nutrition, cosmetology, and the food industry^14–16^. The antioxidant activities of polyphenols can be evaluated in vitro and in vivo using simple experiments^17^.

Solvent extraction is useful for the recovery of antioxidant compounds from plant materials. Extraction yields and antioxidant activities vary depending on the extraction method and solvents used^18^. Polar solvents have gained prominence in the recovery of polyphenols from plant matrices^19^. In particular, aqueous solvent mixtures containing ethanol, methanol, and acetone are effective. Ethanol has gained recognition as a suitable solvent for extracting polyphenols and is safe for human consumption^11^. Conversely, methanol is efficient in extracting low-molecular-weight polyphenols^20^, whereas aqueous acetone has been identified as more suitable for the extraction of greater-molecular-weight flavanols^12^.

Despite the well-established historical uses of *F. communis*, further scientific studies are needed to explore the activities of its extracts. To the best of our knowledge, comprehensive investigations on the biological activity of wild Moroccan *F. communis* fruits are yet to be published. This highlights the novelty and significance of our research, addressing critical knowledge gaps by examining the biochemical constituents of *F. communis* and investigating the effects of various solvents (methanol, ethanol, water, acetone, hexane, ethyl acetate, and chloroform) on antioxidant activity. The antioxidant, antibacterial, and antifungal activities of wild Moroccan *F. communis* L. were evaluated against multidrug-resistant pathogenic strains.

## Methods

### Plant material

*Ferula communis* was harvested at the end of January from Sefrou Province, Morocco (33.6957°N, 4.3716°W) and subsequently identified by Professor Bari Amina, a botanist in the Department of Biology, Faculty of Sciences Dhar El Mehraz, Sidi Mohamed Ben Abdellah University, where a voucher specimen (FC0522) was deposited at the herbarium. Notably, no approval was required to collect *F. communis* from Morocco for research purposes. The fruits used in this study were dried for 15 d in ambient air in the laboratory.

### Extract preparation

The dried fruits (2 g) of *F. communis L.* were powdered using an electric mixer (Blender Waring^®^) and mixed with 20 mL of solvent. Various solvents including methanol, ethanol, water, hexane, acetone, ethyl acetate, and chloroform were used individually. Macerates were filtered through Whatman No. 1 filter paper (Merck KGaA, Darmstadt, Allemagne), and the obtained extracts were stored in sterile sample tubes at 4 °C.

### High-performance liquid chromatography with diode-array detection analysis

Extracts of *F. communis* were prepared at concentrations of 50 mg/mL and filtered through 0.45 μm microfilters. Phenolic compounds were characterized using high-performance liquid chromatography (HPLC) connected to a UV detector (210–400 nm). Notably, 20 μL of extract was injected through a (C18) reverse phase column (250 × 4.6 mm, 5 μm) with an elution gradient of A (water/0.5% phosphoric acid) and B (methanol) at a flow rate of 1 mL/min. Compounds were identified by comparing retention times with authentic standards. Quantification was performed by comparison with external calibration curves and expressed as mg extract equivalents/100 g of dry matter. Analyses were performed in triplicate, and all calibration curves showed good linearity (R^2^ > 0.99).

### Antioxidant activity

#### 2,2-Diphenyl-1-picrylhydrazyl free radical scavenging activity

The total free radical scavenging capacity of various extracts of *F. communis* fruit was estimated according to a previously reported method^14^. Extracts (50 µL) at concentrations ranging from 12.5 to 50 mg/mL were added to 825 μL of ethanolic 2,2-diphenyl-1-picrylhydrazyl (DPPH). The mixture was shaken vigorously and maintained at room temperature in the dark for 1 h. The absorbance of the DPPH radical only served as the blank. All measurements were performed in triplicate. The ability to scavenge DPPH radicals was calculated using Eq. (1):

1 $$\mathrm{Inhibition }(\mathrm{\%}) =\frac{\mathrm{Abs \; control}-\mathrm{Abs\; sample}}{\mathrm{Abs \;control}}\times 100$$

#### 2,2-Azinobis (3-ethyl-benzothiazoline-6-sulfonic acid) radical scavenging activity

Free radical scavenging activity of wild *F. communis* fruit extracts were determined using an 2,2ʹ-azino-bis(3-ethylbenzothiazoline-6-sulfonic acid (ABTS) free radical cation decolorization assay^15^. Briefly, 50 μL of various dilutions of each ethanolic extract or gallic acid (used as a positive control) were added to 825 μL of ABTS radical cation solution. Thereafter, the solutions were incubated at room temperature in the dark for 6 min. Absorption was measured at 734 nm using a UV/Vis spectrophotometer. Absorption of a blank sample containing the same amounts of ethanol and ABTS solution served as the negative control. The inhibition (%) of absorbance was calculated using Eq. (1), and IC_50_ values were determined graphically and expressed as mg ATBS equivalents/mL. All analyses were performed in triplicate.

#### Reducing power

Reducing power (RP) was determined by combining 50 μL of each *F. communis* fruit extract with 200 μL of 0.2 M sodium phosphate buffer (pH 7.6) and 200 μL of 1% potassium ferricyanide^16^. The mixture was stored at 50 °C for 20 min. Two hundred microliters of 10% trichloroacetic acid was added, and the mixture was centrifuged at 2.217×*g* for 10 min. The supernatant was mixed with 600 μL of distilled water and 120 μL of 0.1% ferric chloride. Ascorbic acid (0.073–1.238 mg/mL) was used as a standard to obtain a calibration curve (R^2^ = 0.9996). A graph was plotted using the absorbance at 700 nm versus sample concentration. RP is defined as the extract concentration yielding 0.5% absorbance (EC_50_) and expressed in mg/mL. All measurements were performed in triplicate.

#### Total antioxidant capacity

The total antioxidant capacity (TAC) of extracts were evaluated using the phosphomolybdenum method^17^. Briefly, 50 µL of various extracts was mixed with 1 mL of reagent solution (sulfuric acid 0.6 M, 28 mM sodium phosphate, and 4 mM ammonium molybdate). The absorbance of the sample was recorded against a blank at 695 nm after 90 min of incubation in a water bath at 95 °C using a Perkin Elmer Lambda 40 UV/Vis spectrophotometer. Results are expressed in mg of ascorbic acid equivalents (AAE) per g of dry plant (mg AAE/g DW), as the mean of three triplicate ± standard deviation (SD).

### Total polyphenols and flavonoids in *F. communis*

#### Total phenolic content

The total phenolic content (TPC) was determined using a colorimetric method based on the Folin–Ciocalteu reagent^18^. First, 50 μL of gallic acid extract or standard solution was mixed with 450 μL of Folin–Ciocalteu reagent solution (10%), shaken on a vortex, and incubated for 5 min. Thereafter, 450 μL of a Na_2_CO_3_ solution (75 g L^−1^) was added, and the mixture was shaken again. Following a 2 h incubation at room temperature (25 + 1 °C), absorption was measured at 760 nm using a spectrophotometer. The gallic acid calibration curve displayed high linearity with a standard curve equation (R^2^ = 0.9994). The results are expressed in mg gallic acid equivalent (GAE)/g DW. All analyses were performed in triplicate.

#### Flavone/flavanol content

An aliquant of 500 μL of extracts or quercetin, used as the positive control, was mixed with 500 μL of AlCl_3_ (2%). After incubation for 1 h at ambient temperature, the absorbance of the mixture was measured at 420 nm using a UV/Vis spectrophotometer. Quercetin (0.003–0.5 mg/L) was used to obtain a standard curve (R^2^ = 0.9998), and the results were expressed as milligrams of quercetin equivalent (QE) per gram of sample (mg QE/g). All tests were performed in triplicate^19^.

### Antimicrobial activity of the ethanol, acetone, and water extracts of *F. communis*

#### Microbial strains

The antimicrobial activities of *F. communis* extracts in ethanol (EtOH), acetone (AcE), or water (AqE) were determined against four fungal strains, including *Candida albicans* (ATCC 10231), *Aspergillus niger* (MTCC 282), *Aspergillus flavus* (MTCC 9606), and *Fusarium oxysporum (*MTCC 9913), and four bacterial strains, including *Staphylococcus aureus* (ATCC 6633), *Escherichia coli* (K12), *Bacillus subtilis* (DSM 6333), and *Proteus mirabilis* (ATCC 29906). All microbial strains were provided by the Laboratory of Biotechnology, Environment, Agri-Food, and Health, Faculty of Sciences, Dhar El Mahraz, Sidi Mohammed Ben Abdellah University, Fez, Morocco.

#### Assessment of antimicrobial activity

The antimicrobial activities of the aqueous, ethanol, and acetone extracts of *F. communis* were evaluated using the disk diffusion method^11^. Briefly, Petri dishes containing Mueller–Hinton (MH) culture media and malt extract (ME) were individually inoculated with each of the four bacterial strains in combination with *C. albicans*, using the double layer method. Ten-fold dilutions were prepared in sterile saline (0.9% NaCl) from fresh cultures grown in MH and ME media, reaching a turbidity of 0.5 McFarland (10^6^–10^8^ CFU/mL). Thereafter, 100 µL was added to tubes containing 5 mL soft agar (0.5% agar), and the inoculated tubes were placed into Petri dishes containing MH and EM media. Whatman no. 4 filter paper discs (6 mm in diameter) were impregnated with 20 μL of the AqE, EtOH, and AcE extracts of *F. communis*. For *A. niger*, *A. flavus*, and *F. oxysporum*, the antifungal activity was evaluated by confrontation in ME medium. To assess the efficacy of the negative and positive controls, *F. communis* extracts containing the conventional antibiotics, oxacillin for bacterial strains and fluconazole for fungal pathogens, were tested in a similar manner. Petri dishes inoculated with bacteria and fungi were incubated at 37 °C and 30 °C, respectively^20^. The diameters of inhibition and inhibition percentages were calculated 24 h post-inoculation (hpi) for the bacterial strains, 48 hpi for *C. Albicans*, and 7 d post-inoculation for *F. oxysporum*, *A. niger*, and *A. flavus*^12,21^.

### Minimum inhibitory concentration

The minimum inhibitory concentration (MIC) of the AqE, EtOH, and AcE extracts of *F. communis* against the four bacterial and four fungal pathogens were determined using the microdilution method described by Sarker et al.^22^. Microplates were prepared under aseptic conditions, and each sterile 96-well microplate was labeled. Thereafter, 100 µL of EtOH, AcE, or AqE extracts of *F. communis* in 2% DMSO was pipetted into the first column of the plate. All other wells contained 50 µL sterile MH for bacterial pathogens and 50 µL of sterile EM for fungal pathogens. Serial dilutions were made using a multichannel pipette; finally, 40 µL of microbial suspension of each strain (10^6^ to 10^8^ CFU/mL) was added to each well. After a 24-h incubation period for bacteria, 48-h period for *C. albicans*, and 7-d period for *A. niger, A. flavus*, and *F. oxysporum*, the MIC endpoint was quantified calorimetrically (TTC, 0.2% (w/v))^23^.

### Molecular docking

To elucidate the antioxidant, antibacterial, and antifungal effects of *F. communis, m*ajor compounds in their extracts, including syringic acid (C7), 3-hydroxybenzoic acid (C8), and p-coumaric acid (C12), were docked to the active sites of NADPH oxidase, FimH lectin, and CYP51 proteins encoded in the Protein Data Bank (PDB) using 2CDU, 4XO8, and 5TZ1, respectively. Molecular docking was performed via the following steps: (1) Three-dimensional structures of targeted proteins were extracted from the Research Collaboratory for Structural Bioinformatics (RCSB) PDB basis (https://www.rcsb.org/); (2) Proteins in PDB files were prepared using Discovery Studio 2021 software (https://www.3ds.com/products-services/biovia/products/molecular-modeling-simulation/biovia-discovery-studio/)^24,25^, removing all co-crystallized ligands and water molecules, and adding the charges of Gasteiger; (3) The examined ligands (C7, C8, and C12) and the prepared proteins were converted from PDB to PDBQT files using Autodock software version 4.2 (10.1186/1758-2946-3-12)^26^; (4) Grid boxes were centralized on each targeted protein, maximizing dimensions in three directions (OX, OY, and OZ) with a spacing of 0.375 Å, as shown in Table 1. The produced complexes were visualized using Discovery Studi 2021 software^27^.

**Table 1:** Grid box coordinates for three targeted proteins.

Protein name	Protein code	Protein type	Organism	Grid centers
NADPH oxidase	2CDU.pdb	Oxidoreductase	*Fructi lactobacillus* sanfranciscensis	OX = 10.201, OY = 0.675, OZ = 6.149
FimH lectin	4X08.pdb	Cell adhesion	*Escherichia coli* K-12	OX = -18.429, OY = -6.08, OZ = 7.461
Sterol 14-alpha demethylase (CYP51)	5TZ1.pdb	Oxidoreductase inhibitor	*Candida albicans*	OX = 62.614, OY = 66.604, OZ = 2.766

### Statistical analyses

All results are expressed as the mean of triplicate experiments ± SD. The significance of the difference between means was tested using a two-way analysis of variance (ANOVA). Tukey’s multiple range tests (*p* < 0.05) were performed using GraphPad Prism 8.0.1 (Graph Pad Software Inc., San Diego, United States), and correlations were added using Origin Pro-2023.

### Plant collection approval

No approval is needed to collect *Ferula communis* in Morocco for research purposes.

### IUCN policy statement

The collection of plant material complies with relevant institutional, national, and international guidelines and legislation.

## Results and discussion

### Extraction yield

The water/raw material ratio is a critical factor affecting extraction yield of active components from plant materials. Furthermore, the recovery of phenolic and flavonoid compounds is considerably influenced by polarity^28^. The purpose of the experiments in this study was to assess the effect of solvents on the extraction yield.

The results presented in Table 2 indicate that polar protic solvents had better extraction yields. Among all solvents used, ethanol and methanol achieved significantly higher extraction yields, with rates of 15.25 ± 1.0% and 15 ± 1.2%, respectively. This suggested that ethanol and methanol are optimal solvents for maximizing the extraction yield of *F. communis* fruit, followed by water, acetone, and ethyl acetate with yields of 7.9 ± 0.25%, 2.6 ± 0.05%, and 2.3 ± 0.02%, respectively. However, hexane and chloroform achieved the lowest yields, with rates of 0.9 ± 0.012% and 0.6 ± 0.03%, respectively.

**Table 2:** Extractive yields of *F. communis* fruit extracts (% dry weight).

	HE	ChE	AcE	AEE	EtOH	MeOH	AqE
FC-Ext (%)	0.9 ± 0.012	0.6 ± 0.03	2.6 ± 0.05	2.3 ± 0.02	15.25 ± 1.0	15 ± 1.2	7.9 ± 0.25

HE, hexane extract; ChE, chloroform extract; AcE, acetonic extract; AEE, ethyl acetate extract; EtOH, ethanol extract; MeOH, methanol extract; AqE, aqueous extract; FC-Ext, *F. communis* extract.

The polarity of the ethanol system is highly stable for the extraction of flavonoids and betacyanin glycosides, such as isorhamnetin-3-O-rutinoside, betanin, isobetanin, and betanidine, and polar components, such as phospholipids, polysaccharides, and lipoproteins^20^. Acetone and water are common extractants of proanthocyanidins and tannins^12^.

Our experiments show that the selection of an appropriate extraction solvent depends on the type and polarity of the constituents in the plant material.

### HPLC analysis

**Figure 1:**
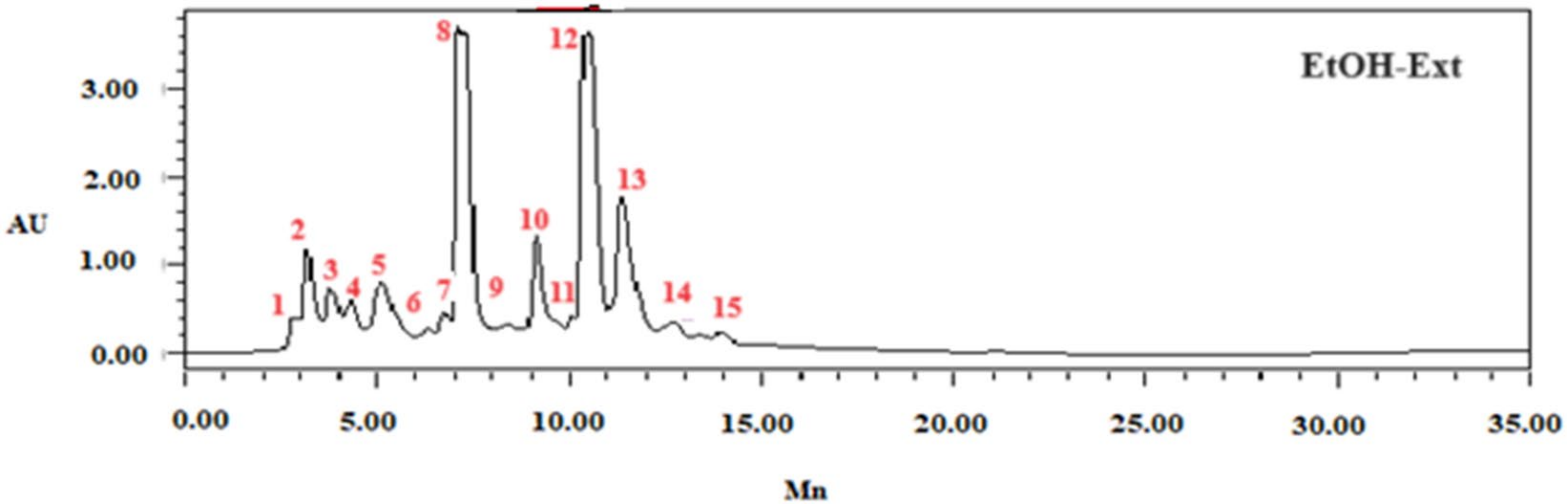
HPLC–DAD chromatogram of *F. communis* extract fruits (FC-Ext) at 320 nm using the following standards: Gallic acid (**1**), Caffeic acid (**2**), Catechin (**3**), 4-Hydroxybenzoic acid (**4**), Catechin hydrate (**5**), Succinic acid (**6**), Syringic acid (**7**), 3-hydroxybenzoic acid (**8**), Naringin (**9**), Cinnamic acid (**10**), Ferulic acid (**11**), *p*-Coumaric acid (**12**), Sinapic acid (**13**), Quercetin 3-*O*-β-d-glucoside (**14**), Rutin (**15**).

Fifteen phenolic compounds, including gallic acid, caffeic acid, catechin, 4-hydroxybenzoic acid, catechin hydrate, succinic acid, syringic acid, 3-hydroxybenzoic acid, naringin, cinnamic acid, ferulic acid, *p*-coumaric acid, sinapic acid, quercetin 3-*O*-β-d-glucoside, and rutin (Fig. 1), were identified and quantified using HPLC–DAD. In a previous study, various phenolic compounds, such as resorcinol, ferulic acid, syringic acid, and coumarin, are identified as the predominant components in *F. communis* fruits from various geographical origins. Compounds were identified according to relative retention times based on reference standards and reported as percentage areas (Table 3)^7^.

**Table 3:** HPLC analysis compounds in *Ferula communis* fruit extracts.

Peak	Standards	Formula	Rt (min)	%Area	λ_max_
1	Gallic acid	C_7_H_6_O_5_	2.895	0.5	280
2	Caffeic acid	C_9_H_8_O_4_	3.174	4.14	320
3	Catechin	C_15_H_14_O_6_	3.749	2.57	254
4	4-Hydroxybenzoic acid	C_7_H6O_3_	4.322	1.73	254
5	Catechin hydrate	C_15_H_14_O_6_	5.093	5.11	280
6	Succinic acid	C_4_H_6_O_4_	6.744	0.86	280
7	Syringic acid	C_9_H_10_O_5_	7.103	12.87	250
8	3-hydroxybenzoic acid	C_7_H_6_O_3_	7.211	17.34	320
9	Naringin	C_27_H_32_O_14_	8.398	0.76	280
10	Cinnamic acid	C_9_H_8_O_2_	9.126	6.84	280
11	Ferulic acid	C_10_H_10_O_4_	10.066	0.9	320
12	*p*-coumaric acid	C_9_H_8_O_3_	10.461	29.42	320
13	Sinapic acid	C_11_H_12_O_5_	11.328	15	320
14	Quercetin 3-*O*-β-d-glucoside	C_21_H_20_O_12_	12.668	1.53	350
15	Rutin	C_27_H_30_O_16_	13.909	0.43	254

In a study focusing on *F. communis* rhizomes and employing a bioautography-guided isolation technique, three antibacterial sesquiterpenes were successfully isolated and characterized. These compounds were identified as daucane 14-(O-hydroxycinnamoyloxy)-dauc-4,8-diene, 2a-acetyl-6a-(benzoyl) jaeschkeanadiol, and 2a-acetyl-6a-(p-anisoyl) jaeschkeanadiol^29^. Additionally, ferulenol, a constituent of *F. communis*, has garnered attention owing to its various pharmacological effects, including antimicrobial, anticoagulant, antiproliferative, and antiappetitant activities. Recently, Gliszczynska et al., explored sesquiterpene coumarins, focusing on ferulenol, as promising lead compounds for drug discovery^30^. Notably, this diverse array of secondary metabolites substantially contributes to the antioxidant capabilities of *F. communis* while simultaneously conferring a broad spectrum of pharmacological and biological functionalities^31^. Phenolic acids, known for their various biological applications, are the primary polyphenols produced by plants and serve as precursors for bioactive molecules used in the therapeutic, cosmetic, and food industries. The health-promoting effects of these phenolic compounds are diverse and may include anticancer, anti-inflammatory, antioxidant, antimicrobial, and anti-aging properties^32^.

### Total phenolic content

**Figure 2:**
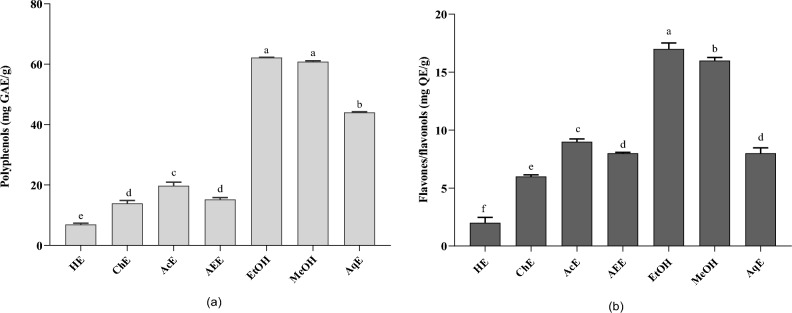
Effect of different solvent extractions on the (**a**) polyphenol content; (**b**) flavone/flavanol content; results sharing the same letter in the same test are not significantly different according to Tukey’s multiple range test (p < 0.05). Data are the means of three replicates. HE, Hexane extract; ChE, chloroform extract; AEE, ethyl acetate extract; EtOH, ethanolic extract; MeOH, methanol extract; AqE, aqueous extract.

Phenolic compounds are the primary phytochemicals responsible for the biological activities of *Ferula*^3^. Moreover, the solvent influences the quantity and selectivity of the extracted components^33^. Phenolic compounds and other phytochemicals containing hydroxyl groups are primarily soluble in polar solvents^34^. As shown in Fig. 2a, our results present the effects of different extraction solvents (ethanol, methanol, water, hexane, ethyl acetate, and chloroform) on the total polyphenol content of *F. communis*. The ethanol extract exhibited the highest total polyphenol content (62.20 ± 0.11 mg GAE/g DW), followed by the methanol (60.82 ± 0.32 mg GAE/g DW) and aqueous (44.04 ± 0.22 mg GAE/g DW) extracts. Acetone, ethyl acetate, chloroform, and hexane showed lower values at 19.76 ± 1.19, 15.24 ± 0.60, 13.94 ± 0.92 and 6.93 ± 0.43 mg GAE/g DW, respectively. Comparing the TPC of the solvents, the order established was: ethanol > methanol > water > acetone > ethyl acetate > chloroform > hexane. The extract of Tunisian *F. communis* fruit exhibited the highest TPC (422 mg GAE/g DW), followed by flowers (207.21 mg GAE/g DW) and stems (129.86 mg GAE/g DW)^35^. In addition, Gamal and Atraimki quantified the total phenolics in n-butanol and ethyl acetate extracts (44.7–55.8 mg GAE/g DW)^36^.

The extraction efficiencies varied considerably among the solvents, resulting in differences in the concentrations of total phenolics. The TPC of the extracts is influenced by the polarity of the extracting solvent and the solubility of chemical constituents in the solvent^37^. Solvents with greater polarity extracted more TPC^29^. Thus, more polar solvents such as ethanol, methanol, and acetone extracted more TPC. Ethanol extracts have the highest TPC, followed by methanol and acetone extracts^17^. This is consistent with a previous study, in which methanol and ethyl acetate extracted greater amounts of phenolic compounds than the other two^37^. These results confirm the critical role of extraction solvents in modifying the TPC recovery from plant material. Furthermore, based on the above results, it is clear that the ethanol and methanol extracts of wild *F. communis* fruit examined in this study exhibited comparable or greater efficiency of TPC extraction than previously reported.

### Total flavonoid content

The total flavonoid content (TFC) from *F. commnis* exhibited a trend similar to that of TPC (Fig. 2b). The ethanol extract of *Ferula* exhibited the highest extraction yields of flavonoids (17.09 mg QE/g DW), followed by the methanol extract with 16.91 ± 0.26 mg QE/g DW, and the acetone extract (9.31 ± 0.24 mg GAE/g DW). Aqueous, ethyl acetate, chloroform, and hexane showed lower extraction yields with values of 9.31 ± 0.24, 8.97 ± 0.47, 8.68 ± 0.08, 6.23 ± 0.13, and 2.39 ± 0.47 mg GAE/g DW, respectively. Owing to their polarities, the extraction of TFC significantly varied among the solvents. For the TFC, the extracts were classified in decreasing order of efficiency: ethanol > methanol > acetone > water > ethyl acetate > chloroform > hexane. The methanol extracts of various aerial parts of *F. communis L.* showed the highest concentration in flowers, followed by fruits and stems, with values of 48.77, 14.23, and 13.37 mg QE/g DW, respectively. Our results, reflecting varying TFC amounts extracted by different solvents, can explain the presence of diverse groups of soluble flavonoids in *F.* communis fruits with distinct polarities. In addition, reported variations in bioactive compound values for *Ferula* in the literature may stem from factors such as varietal differences, environmental conditions, agronomic practices, and analytical methods.

### Antioxidant activity of *F. communis*

**Figure 3:**
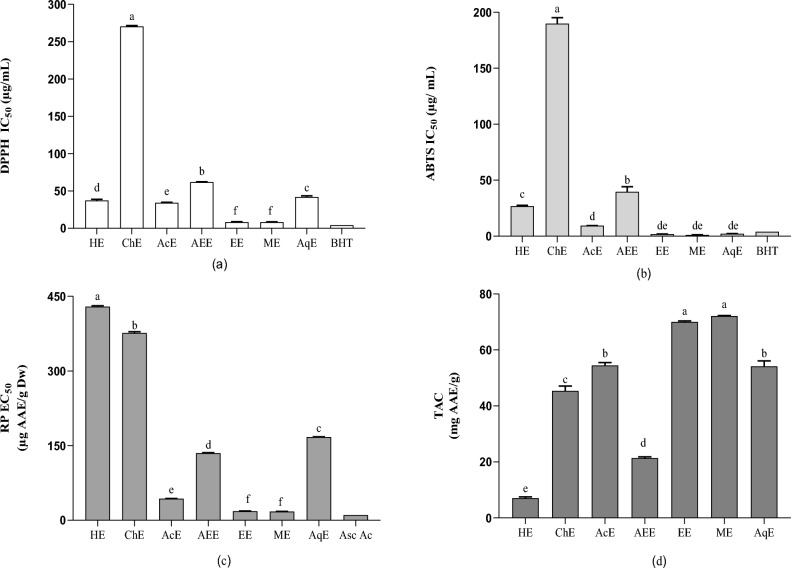
Antioxidant activities: (**a**) IC_50_ of the DPPH test; (**b**) IC_50_ of the ABTS test; (**c**) EC_50_ of reducing power; (**d**) the content of total antioxidant capacity (TAC). Each response is the average of triplicate with standard error. Values in the same column followed by different letters are significantly different according to Tukey’s multiple range test (p < 0.05). HE, Hexane extract; ChE, chloroform extract; AEE, ethyl acetate extract; EtOH, ethanolic extract; MeOH, methanolic extract; AqE, aqueous extract; IC_50_, the extract concentration provides 50% inhibition; DPPH, 2,2‐diphenyl‐1‐picrylhydrazyl; ABTS, 2,2′-azino-bis (3-ethylbenzothiazoline-6-sulphonic acid); TAC, total antioxidant capacity.

Antioxidant activity was not assessed using a single antioxidant test^30^; rather, several in vitro procedures, including the DPPH, ABTS, RP, and TAC assays (Fig. 3), were used to assess antioxidant activity. As antioxidant test models vary in different respects, it is difficult to directly compare results^9,34^. Therefore, performing several screens using ex vivo models and in vivo studies is essential^31^. DPPH is a nitrogenous organic radical with free electrons, and is an easy and valuable spectrophotometric method for screening or measuring antioxidant activity^38^. When antioxidants react with DPPH, stable free radicals pair with hydrogen donors and are reduced to DPPH-H^28^. ABTS is a target molecule used to assess the reactivity of antioxidant samples in the presence of peroxides^15^. The radical cation of ABTS (ABTS^+^) is synthesized by oxidizing ABTS with potassium permanganate, potassium persulfate, or 2,2′-azo-bis (2-amidinopropane)^39^. The ABTS assay reduces labor time, material cost, and sample volume, while decreasing color^40^. The RP assay is based on the principle that substances with a reducing potential react with potassium ferricyanide (Fe^3+^) to form potassium ferrocyanide (Fe^2+^)^41^, consequently reacting with ferric chloride to form a ferric-ferrous complex with an absorption maximum at 700 nm^42^.

The greatest DPPH scavenging capacity was demonstrated by the methanol extract with an IC_50_ value of 8.09 µg/mL, followed by the ethanol, acetone, and aqueous extracts with IC_50_ values of 8.25, 34.23, and 37.28 μg/mL, respectively. The scavenging efficacy decreased in the following order: ChE < AEE < AqE < AcE < EtOH < MeOH. These results demonstrated that highly polar solvents, including ethanol and methanol, are effective for the extraction of antioxidants with efficient free radical scavenging properties and are more effective than intermediate-polarity solvents, such as acetone and hexane, but not chloroform (Fig. 3a).

The results of this study are consistent with those of previous studies wherein potent antioxidant activities were observed for both ethyl acetate (IC_50_ = 4.4 ± 0.1 μg/mL) and n-butanol extracts^36^. The antioxidant activity assessed by measuring the scavenging power of DPPH on gum resin of the Algerian *F. communis* L. harvested in the Sahara had an IC_50_ of 5.08 mg/mL^43^. Roots of another species, *Ferula gummosis* Boiss, was evaluated for antioxidant activity using the DPPH assay and displayed an IC_50_ = 579.6 ± 19.4 μg/mL^44^. The RP of *F. communis* extracts was highest in the MeOH extract, with an EC_50_ of 17.63 µg, followed by the ethanol, acetone, and ethyl acetate extracts, with EC_50_ values of 17.63, 43.4, and 135 µg AAE/g DW, respectively. Hexane exhibited the lowest RP with an EC_50_ of 429.3 μg AAE/g DW. The high RP of the *F. communis* methanol extract is probably due to clusters of hydroxyls in phenolic compounds, serving as electron donors. Therefore, antioxidants act as both reducers and activators^45^. The RP of a compound can considerably indicate its antioxidant potential^46^. However, no study has evaluated the RP of *F. communis* fruits.

The results of the ABTS assay showed significant variations among the extracts (Fig. 3b). The ethanol extract expressed the highest percentage of ABTS free radical inhibition (IC_50_ = 1.88 ± 0.07 μg/mL). Alternatively, the chloroform extract (ChE) showed the lowest ABTS radical scavenging capacity (IC_50_ = 189.87 μg/mL). The activity (ABTS) results for *F. communis* extracts have not been previously published.

Reduced antioxidant activity, as determined by the TAC assay (Fig. 3d), was similar to the activities determined by the other tests. The MeOH and EtOH extracts exhibited the highest values (72.15 ± 0.11 and 70.04 ± 0.34 mg AAE/g, respectively). In addition, the hexane sample showed the least RP (7.05 ± 0.46 g AAE/g). The methanol, ethanol, acetone, and aqueous extracts showed powerful and significant correlations in the four antioxidant tests (Table 4), possibly related to their phytochemical constituents, primarily phenolic components. These results supported the investigation of the antimicrobial activity of these extracts.

**Table 4:** Pearson correlation coefficients.

	Polyphenols	Flavones/flavanols	DPPH	ABTS	RP	TAC	*S. aureus*	*E. coli*	*B. subtilis*	*P. mirabilis*	*C. albicans*	*A. niger*	*F. oxysporum*
Polyphenols	1	0.916∗∗∗	− 0.482∗∗∗	− 0.494	− 0.524	0.847∗∗∗	0.186∗∗∗	0.304∗∗∗	0.348∗∗∗	0.108∗∗∗	0.427∗∗∗	0.335∗∗∗	0.114∗∗∗
Flavonoids		1	− 0.441∗∗∗	− 0.429∗∗∗	− 0.679∗∗∗	0.855∗∗∗	0.352∗∗∗	0.447∗∗∗	0.480∗∗∗	0.284	0.349	0.470∗∗∗	0.290
DPPH			1	0.990∗∗∗	− 0.039∗∗∗	− 0.170∗∗∗	− 0.321∗∗∗	− 0.340∗∗∗	− 0.342∗∗∗	− 0.300∗∗∗	− 0.392∗∗∗	− 0.342∗∗∗	− 0.302∗∗∗
ABTS				1	0.019∗∗∗	− 0.209∗∗∗	− 0.329∗∗∗	− 0.337∗∗∗	− 0.335∗∗∗	− 0.315∗∗∗	− 0.473∗∗∗	− 0.336∗∗∗	− 0.316∗∗∗
RP					1	− 0.747∗∗∗	− 0.308∗∗∗	− 0.310∗∗∗	− 0.305∗∗∗	− 0.299∗∗∗	− 0.391∗∗∗	− 0.307∗∗∗	− 0.300∗∗∗
TAC						1	0.449∗∗∗	0.499∗∗∗	0.511∗∗∗	0.407∗∗∗	0.554∗∗∗	0.508∗∗∗	0.411
*S. aureus*							1	0.973∗	0.946∗	0.990∗∗∗	0.729∗∗∗	0.955∗∗∗	0.991∗∗∗
*E. coli*								1	0.995	0.930∗	0.748∗∗∗	0.998∗∗∗	0.934∗∗∗
*B. subtilis*									1	0.890∗∗∗	0.744∗∗∗	1.000∗∗∗	0.895∗∗∗
*P. mirabilis*										1	0.698∗∗∗	0.903∗∗∗	1.000
*C. albicans*											1	0.746∗∗	0.701∗∗
*A. niger*												1	0.908
*F. oxysporum*													1

Statistical significance, ∗p < 0.05; ∗∗p < 0.01; ∗∗∗p < 0.001.

### Antimicrobial studies

#### Antibacterial activity

**Figure 4:**
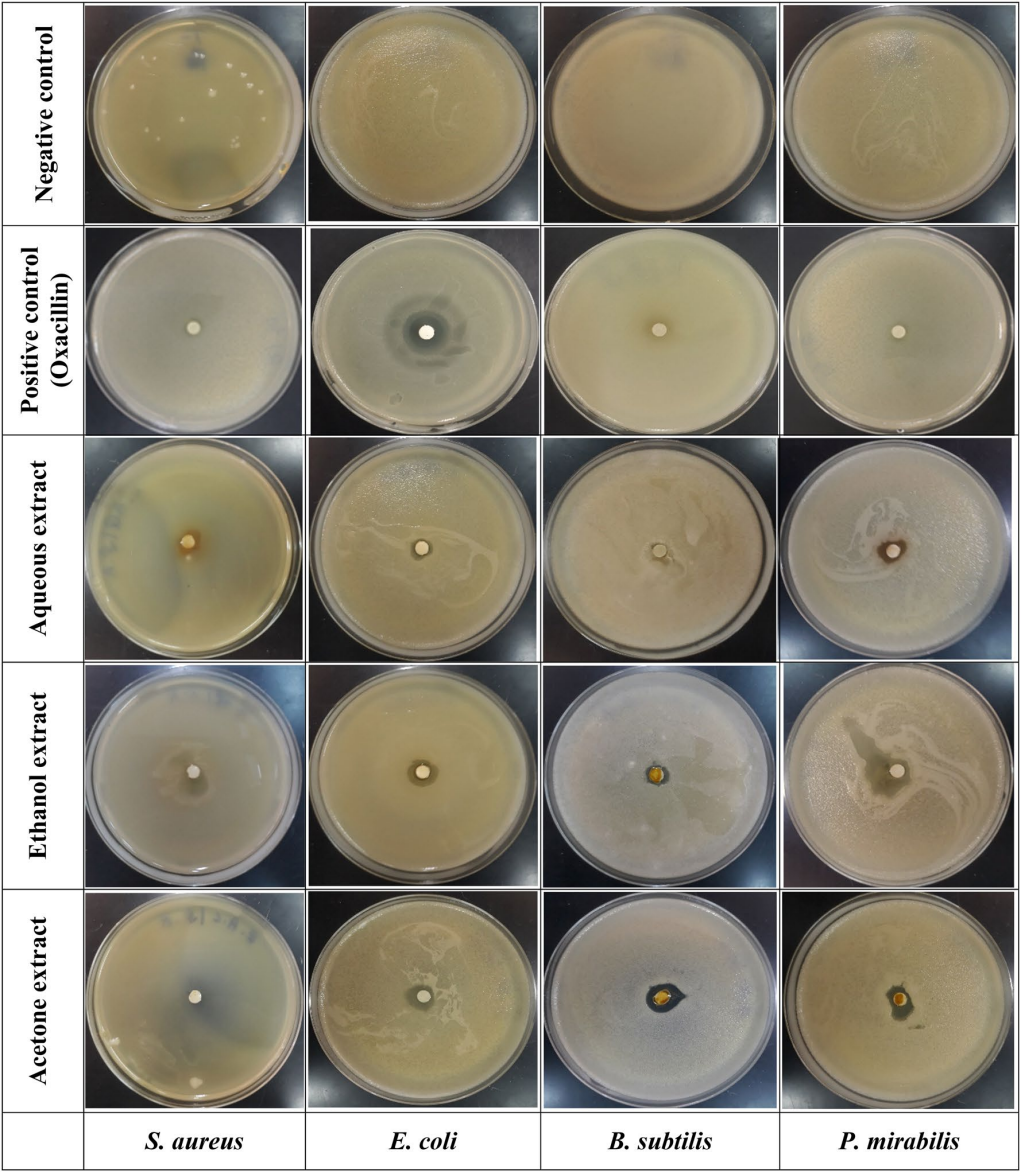
Visual observations of the inhibitory effects of *Ferula communis* extracts against pathogenic bacteria, including *Staphylococcus aureus* ATCC6633, *Escherichia coli* K12, *Bacillus subtilis* DSM6333, and *Proteus mirabilis* ATCC29906.

The antimicrobial activities of *F. communis* extracts, including EtOH, AqE, and AcE, were evaluated against the examined microorganisms. EtOH and AcE extracts exhibited significant antibacterial activity compared to the reference antibiotic, oxacillin, as determined by the inhibition zone diameter and MIC values (Tables 5, Fig. 4). The AcE extract displayed the highest inhibitory activity against *P. mirabilis* with an inhibition diameter of 19.00 ± 1.00 mm and an MIC of 2.50 ± 0.00 mg/mL, followed by *E. coli* with 11.50 ± 1.50 mm of inhibition zone and an MIC of 2.50 ± 0.00 mg/mL. The extract also exhibited inhibitions of 11.00 and 9.00 mm against *S. aureus* and *B. subtilis*, respectively.

**Table 5:** Antibacterial activity of the aqueous, ethanol, and acetone extracts of *Ferula communis.*

		*S. aureus* ATCC6633	*E. coli* K12	*B. subtilis* DSM6333	*P. mirabilis* ATCC29906
Aqueous extract	Antibacterial activity (mm)	0.00 ± 0.00^a^	0.00 ± 0.00^a^	0.00 ± 0.00^a^	0.00 ± 0.00^a^
	MIC (mg/mL)	–	–	–	–
Ethanolic extract	Antibacterial activity (mm)	9.00 ± 0.00^b^	14.00 ± 1.00^b^	13.00 ± 2.00^b^	12.00 ± 1.00^b^
	MIC (mg/mL)	2.50 ± 0.00^B^	0.312 ± 0.00^A^	0.625 ± 0.00^A^	0.625 ± 0.00^A^
Acetone extract	Antibacterial activity (mm)	11.00 ± 1.00^c^	11.50 ± 1.50^c^	9.00 ± 0.00^c^	19.00 ± 1.00^c^
	MIC (mg/mL)	2.50 ± 0.00^B^	2.50 ± 0.00^B^	2.50 ± 0.00^B^	2.50 ± 0.00^B^
Oxacillin	Antibacterial activity (mm)	0.00 ± 0.00^a^	18 ± 0.00^d^	0.00 ± 0.00^a^	0.00 ± 0.00^a^
	MIC (mg/mL)	–	0.312 ± 0.00^A^	–	–

Mean values (± SD, n = 3) with different small letters (for antibacterial activity) and capital letters (for MIC) in the same column are significantly different (two-way ANOVA; Tukey's test, *p* < 0.05). MIC: minimum inhibitory concentration.

The EtOH extract, which contained the largest amounts of TPC and TFC, also exhibited the greatest TAC and was the most effective against all bacteria. The greatest activity against *E. coli*, with an inhibition diameter of 14.00 ± 1.00 mm, an MIC of 0.312 ± 0.00 mg/mL, and the least zone of 9.00 ± 0.00 mm, was observed for *S. aureus*. AcE was more potent than EtOH for both extracts. Both extracts showed bactericidal activity against the four tested bacterial strains, whereas the aqueous extract of *F. communis* did not inhibit any of the examined strains. These differences in inhibition diameters can be attributed to the chemical composition of *F. communis*, and its antibacterial activity may primarily be because of the compounds in these extracts (EtOH, AqE, and AcE). The results showed that the ethanol and acetone extracts inhibited the four bacterial strains, with the ethanol extract having the best antibacterial activity.

Resorcinol, ferulic and syringic acids, and coumarin are renowned for their antibacterial activity. Compared to streptomycin sulfate, the antibacterial activities of the constituents extracted from the rhizome of *F. communis* revealed that the sesquiterpene 14-(hydroxycinnamoyloxy)-dauc-4,8-diene possesses vigorous activity against *S. aureus, B. subtilis, E. faecalis*, and *S. durans*^7^. The 80% ethanol-solubilized fraction of *F. communis* exhibited the greatest antibacterial effects on all assayed bacteria, whereas the aqueous-solubilized fractions did not exhibit any effect on the clinical isolates of *Strep. pyogenes* and *Strep. pneumonia*^47^. Alternatively, the isolated oil of Tunisian *Ferula lutea* was tested for its antimicrobial activity using disc diffusion and microdilution assays against six gram-positive and five gram-negative bacteria and eight species of the genus *Candida*^48^. Oil from flowers of *F. lutea* exhibited antibacterial and anticandidal activity, with an MIC of 39 g/mL against *S. aureus, S. epidermidis*, and *E. coli*, and an MIC of 156 g/mL against *C. albicans*. The type of solvent used considerably affected the antibacterial activity of extracts of *Ferula* inflorescence. These antibacterial compounds were marginally more abundant in gram-negative bacteria than in gram-positive bacteria. This result is likely due to the shape and composition of the cell walls of gram-positive bacteria, which have a thick layer of peptidoglycan with teichuronic acid and glycopolymers such as teichoic acids, making them less sensitive to the action of plant extracts^49^.

#### Antifungal activity

**Figure 5:**
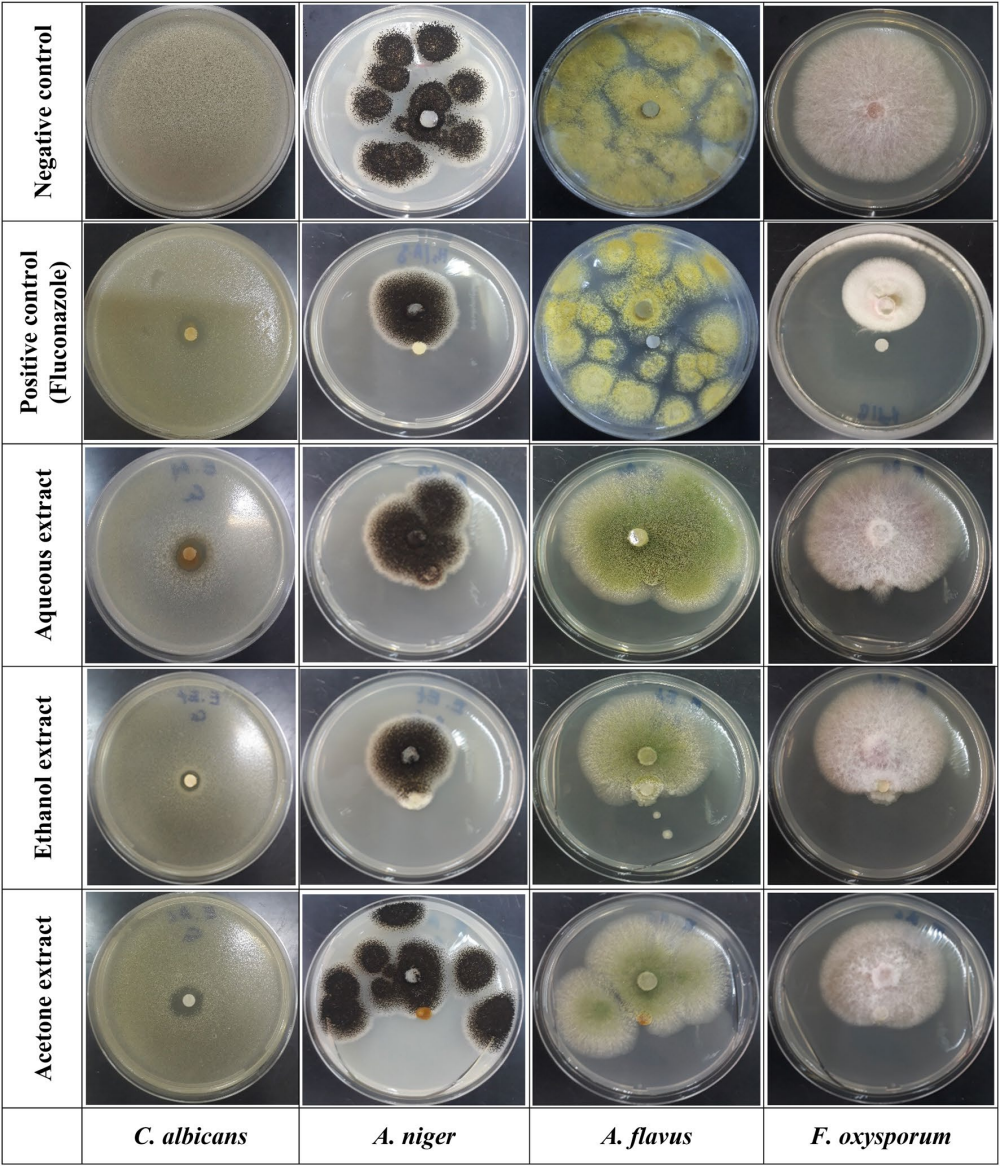
Visual observations of the inhibitory effects of *Ferula communis* extracts against pathogenic fungi, including *Candida albicans* ATCC10231, *Aspergillus niger* MTCC282, *Aspergillus flavus* MTCC9606, and *Fusarium oxysporum* MTCC9913.

In in vitro disk diffusion assays, the antifungal activities of *F*. *communis* extract against *C*. *albicans*, *A*. *niger*, *A*. *flavus*, and *F*. *oxysporum* were compared to that of the fungicide fluconazole. AcE exhibited significant activity against *F*. *oxysporum*, with a percent inhibition of 20.6 ± 1.4% and an MIC value of 5.0 ± 0.0 mg/mL. Additionally, it showed a percentage of inhibition of 19.0 ± 1.0% and an MIC value of 5 mg/mL against *A*. *niger.* The EtOH extract resulted in a small zone of inhibition of only 12.50 ± 2.50 mm) against *C. albicans* with an MIC of 2.50 mg/mL (Table 6, Fig. 5). Additionally, the ethanol extract exhibited an inhibition percentage of 26.0 ± 2.0% and an MIC of 2.5 mg/mL against *A. niger.* The percent inhibition of *F. oxysporum* was 13.3 ± 2.6 with an MIC of 5 mg/mL. The aqueous *F. communis* extract demonstrated antifungal activities against *C. albicans* with a 13.00 ± 1.00 mm of inhibition zone and an MIC of 5 mg/mL for MIC, as this extract has no effect against *A. niger* and *F. oxysporum*. However, the three *F. communis* extracts did not exhibit antifungal effects against *A. flavus*.

**Table 6:** Antifungal activity of the aqueous, ethanol, and acetone extracts of *Ferula communis.*

		*C. albicans* ATCC10231 (%)	*A. niger* MTCC282 (%)	*A. flavus* MTCC9606 (%)	*F. oxysporum* MTCC9913 (%)
Aqueous extract	Antifungal activity (mm)	13.00 ± 1.00^a^	0.00 ± 0.00^a^	0.00 ± 0.00^a^	0.00 ± 0.00^a^
	MIC (mg/mL)	5.00 ± 0.00^A^	–	–	–
Ethanolic extract	Antifungal activity (mm)	14.50 ± 0.50^b^	26.00 ± 2.00^b^	0.00 ± 0.00^a^	13.30 ± 2.56^b^
	MIC (mg/mL)	2.50 ± 0.00^B^	2.50 ± 0.00^A^	–	5.00 ± 0.00^A^
Acetone extract	Antifungal activity (mm)	12.50 ± 2.50^c^	19.00 ± 1.00^c^	0.00 ± 0.00^a^	20.60 ± 1.039^c^
	MIC (mg/mL)	2.50 ± 0.00^B^	5.00 ± 0.00^B^	–	5.00 ± 0.00^A^
Fluconazole	Antifungal activity (mm)	13.00 ± 1.00^a^	20.67 ± 1.52^d^	0.00 ± 0.00^a^	30.67 ± 2.082^d^
	MIC (mg/mL)	7.50 ± 0.00^C^	7.50 ± 0.00^C^	–	7.50 ± 0.00^B^

Mean values (± SD, n = 3) with different small letters (for antifungal activity) and capital letters (for MIC) in the same column are significantly different (two-way ANOVA; Tukey's test, *p* < 0.05). MIC: minimum inhibitory concentration.

Studies on the activity of *F. communis* extracts are scarce. More detailed information on the antimicrobial activities, especially the antifungal properties of *F. communis*, is required. The in vitro antibacterial activities of *F. communis* root extracts on colonies and conidia of *Botryotinia fuckeliana, Penicillium digitatum, P. expansum*, *Monilinia laxa, M. fructigena,* and *Aspergillus spp* were determined^50^; minimal antifungal activity on colony growth was observed, whereas root extracts exhibited the greatest effects on the growth of fungal colonies. However, the root extract was unable to inhibit conidia germination. These results are encouraging because the extract was not a pure product; therefore, the antimicrobial activity may be owing to several compounds related to the presence of bioactive metabolites.

### Correlation

**Figure 6:**
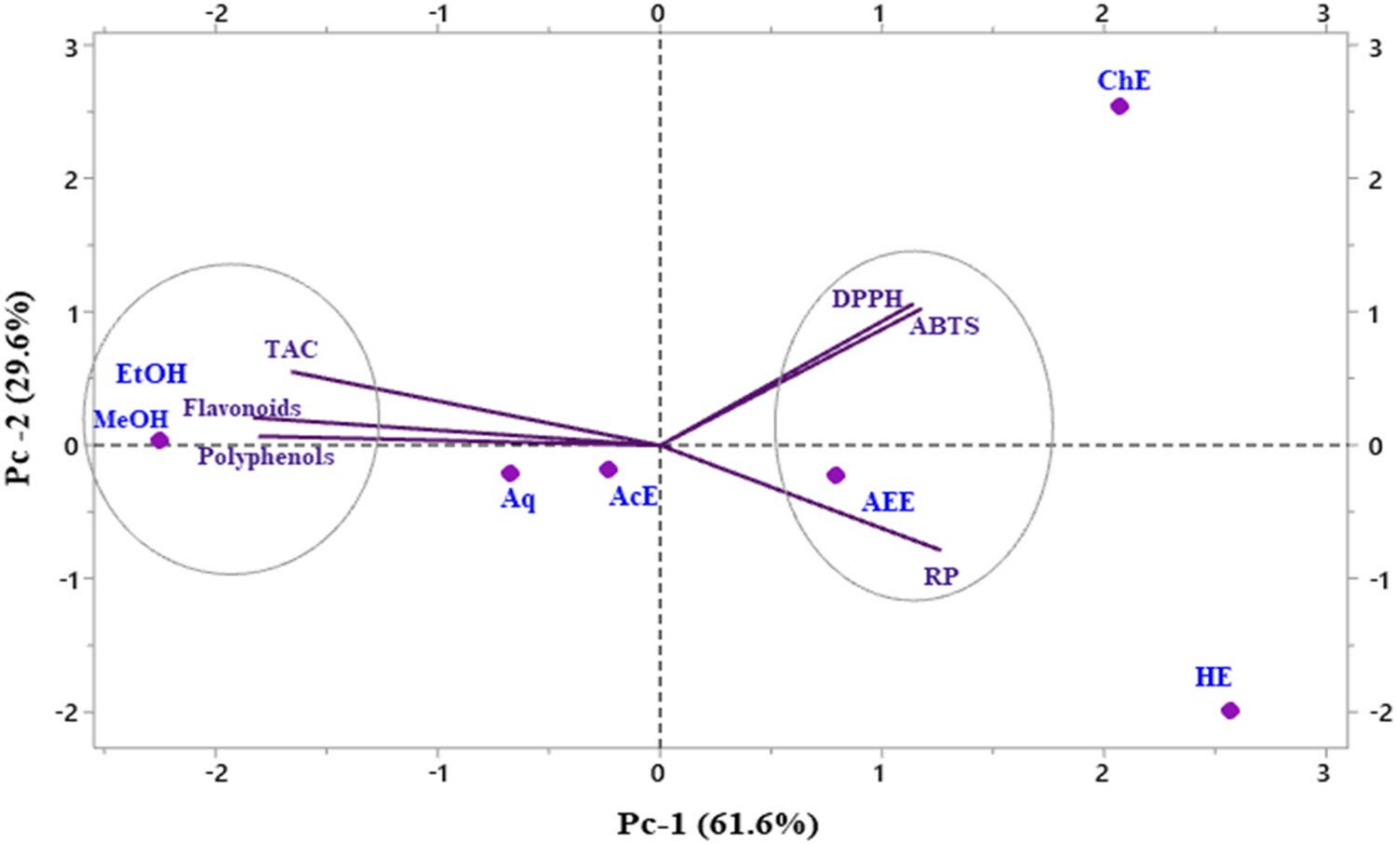
Principal component analysis.

**Figure 7:**
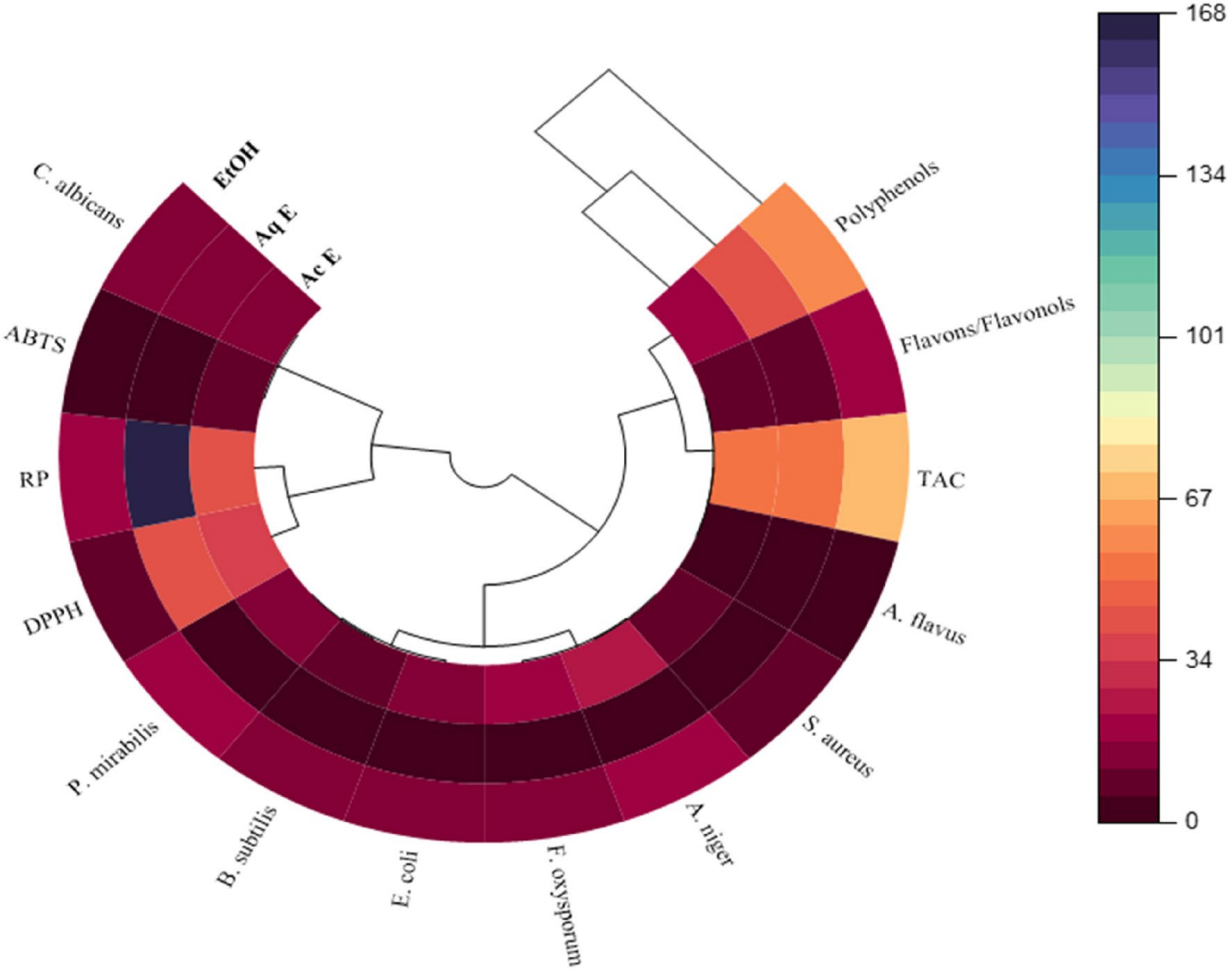
Polar heatmap with dendrogram for antioxidant and antibacterial correlations.

Multivariate analyses, such as principal component analysis (PCA), can reduce a complex dataset, make it more interpretable, and reveal relationships between or among parameters. In the present study, the correlations between antioxidant content, antioxidant activities, and antioxidant and antibacterial activities were examined. PCA results e(Fig. 6) showed that 91.2% of data variability is attributable to the first two principal components, PC1 (Component 1) and PC2 (Component 2), confirming that the correlation test is considered an excellent tool to reveal any relationship between different parameters studied. The first three PCs explained 100% of the data variance, which was sufficient to illustrate all the variables adequately. In this analysis, if two vectors subtend at a slight angle to each other, the two variables they represent strongly correlate. The plots of the scores for PC1 and PC2 showed a strong positive correlation among TPC, TFC, and TAC. The results of the DPPH, ABTS, and RP assays revealed a strong negative correlation between the bioactive content, as represented by the presence of phenolic and flavones/flavanol compounds, and antioxidant activity. Additionally, ethanol and methanol positively and negatively correlated with hexane, respectively. However, a positive correlation between DPPH and ABTS was observed, indicating that the most potent moieties for scavenging DPPH radicals showed the highest ABTS. The ingenious display of the polar heat map revealed a row and column hierarchical cluster structure in the data matrix (similarity/dissimilarity). This made it possible to group different samples based on their similarities. The analysis based on Pearson’s correlation showed lower concentrations in brown and greater concentrations in dark blue (Fig. 7). Plant extracts of the genus *Ferula* have been arranged into two groups, one of which is the ethanol and aqueous extract. The polar heatmap also shows the correlation between the antioxidant content and antioxidant and antibacterial activities.

### Molecular docking simulations

**Figure 8:**
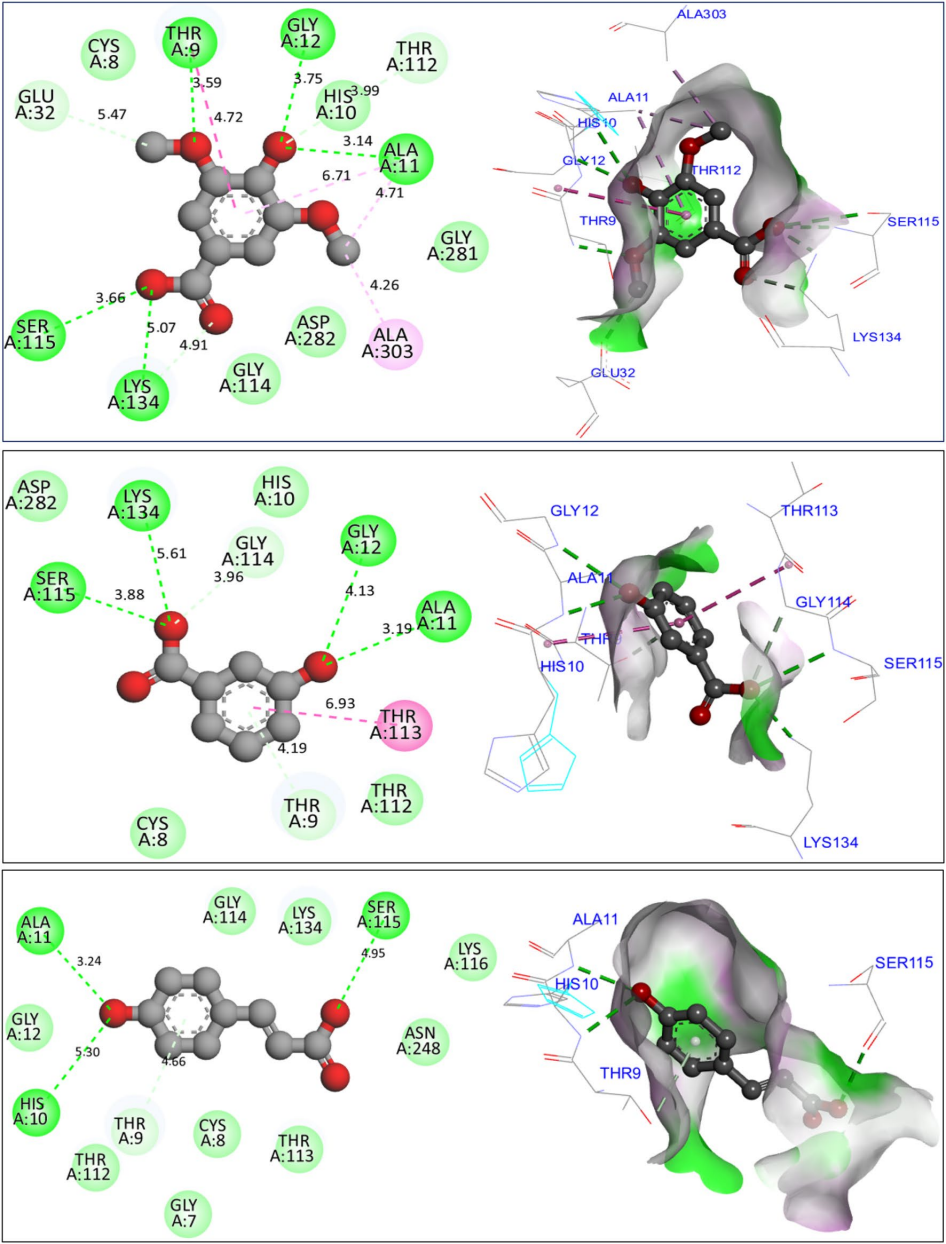
Results of intermolecular interactions between the NADPH oxidase protein from *Lactobacillus sanfranciscensis* (2CDU.pdb) and major compounds of *Ferula communis* (syringic acid, 3-hydroxybenzoic acid, and *p*-coumaric acid) in two and three dimensions.

**Figure 9:**
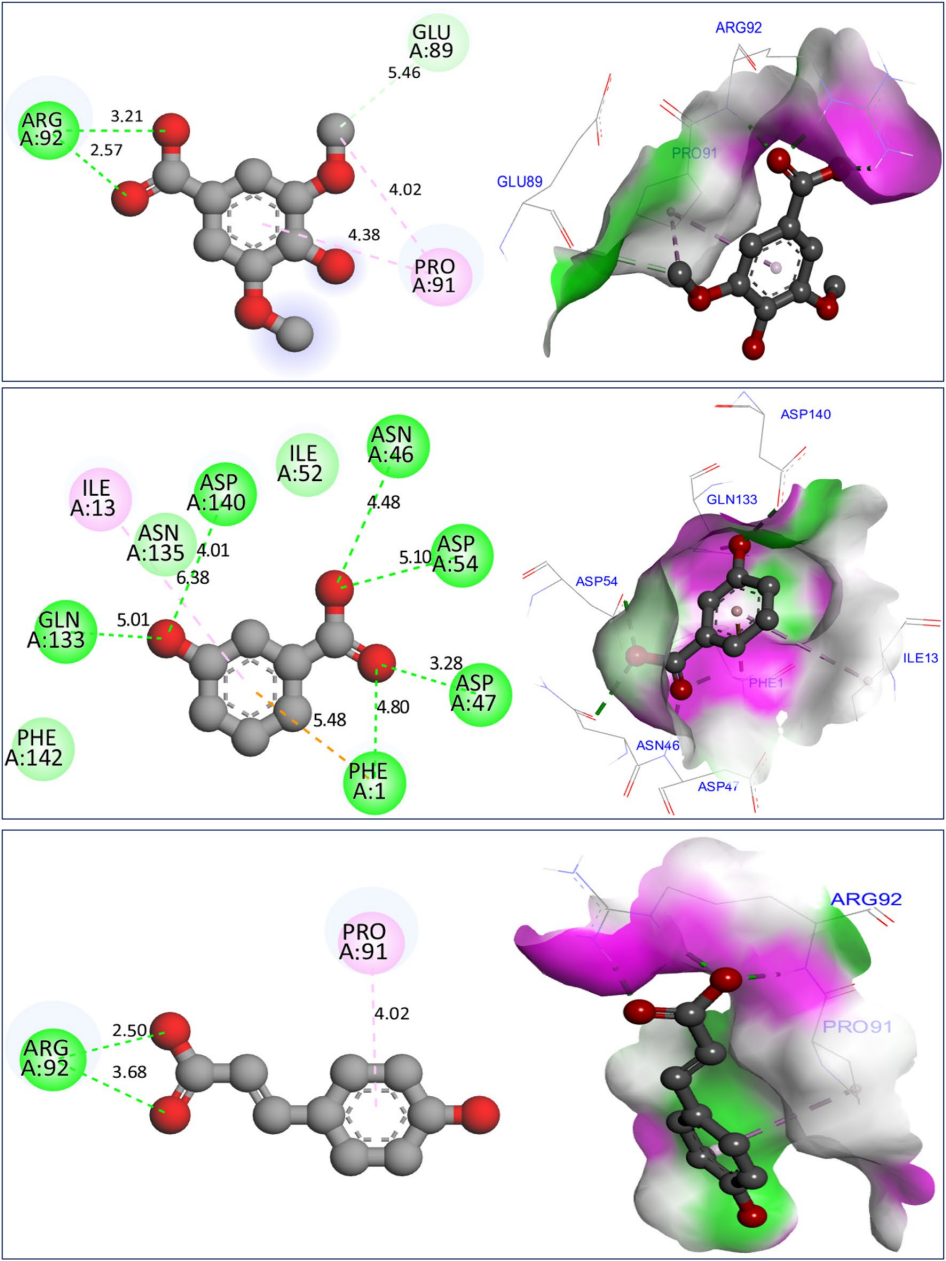
Results of intermolecular interactions between the FimH lectin protein from *Escherichia coli* K12 (4XO8.pdb) and major compounds of *Ferula communis* (syringic acid, 3-hydroxybenzoic acid, and *p*-coumaric acid) in two and three dimensions, produced.

In investigating potential mechanisms of inhibition via antioxidant, antibacterial, and antifungal activities, nine molecular docking simulations were conducted on three targeted proteins with three major compounds of *F. communis*: syringic acid (C7), 3-hydroxybenzoic acid (C8), and *p*-coumaric acid (C12). These compounds were extracted at rates of 12.87, 17.34, and 29.42%, respectively (Fig. 8). Three major compounds were docked to the NADPH oxidase protein from *Lactobacillus sanfranciscensis*. Encoded by 2CDU.pdb, this protein exhibits binding energies of -5.28, -5.76, and 5.65 kcal/mol. It shares various common intermolecular interactions, forming hydrogen bonds with Ser115, Ala11, Lys134, and Gly12 amino acid residues (AAR) in the A chain. Additionally, it forms carbon–hydrogen bonds toward Thr9 AAR. When three major compounds were docked to the FimH lectin protein from *E. coli* K-12, encoded in the PDB by 4XO8.pdb, they formed another family of chemical bonds with binding energies of -5.38, -5.47, and -5.01 kcal/mol. Common bonds included one hydrogen bond with Arg92 AAR and one Pi-Pi alkyl bond with Pro91 AAR. This similarly was observed between syringic acid (C7) and *p*-coumaric acid (C12) (Fig. 9). 3-Hydroxybenzoic acid created another class of chemical bonds, forming six hydrogen bonds with Asp47, Asp54, Asp140, Phe1, Gln133, and Asn46 AAR, in addition to one Pi-alkyl bond with Il13 AAR in the A chain.

**Figure 10:**
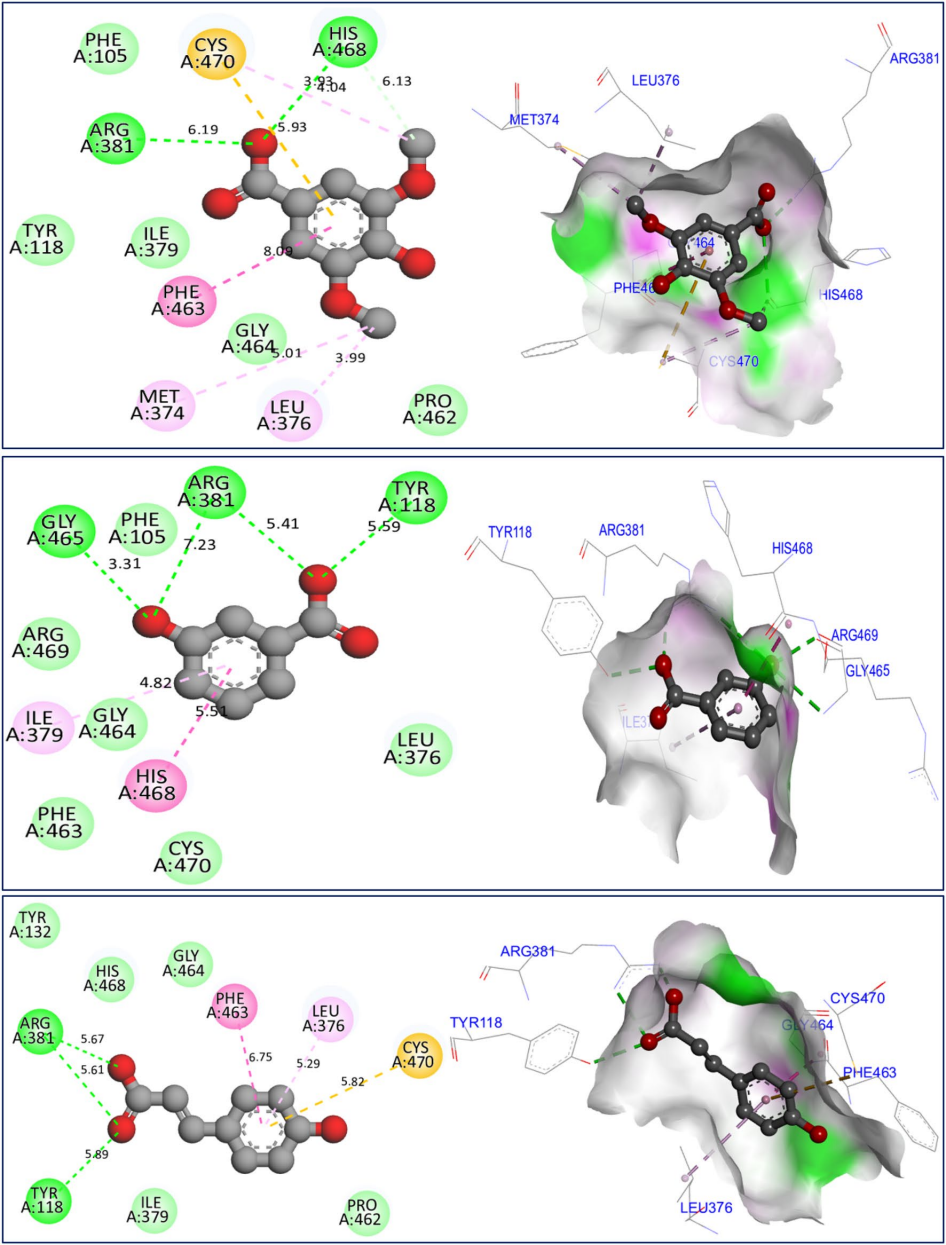
Results of intermolecular interactions between the CYP51 protein from *Candida albicans* (5TZ1.pdb) and major compounds (syringic acid, 3-hydroxybenzoic acid, and *p*-coumaric acid) in two and three dimensions.

**Figure 11:**
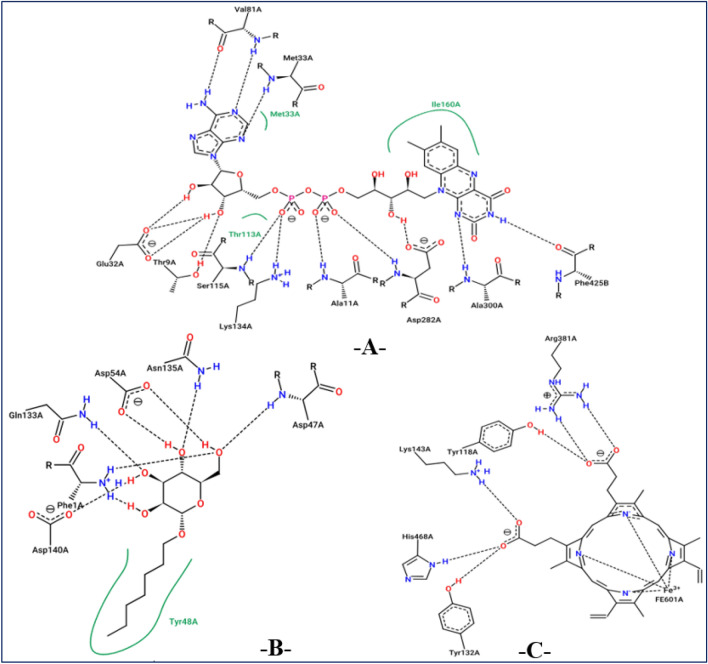
Active sites of antioxidant (2CDU.pdb), antibacterial (4XO8.pdb), and antifungal (5TZ1.pdb) proteins complexed with flavin-adenine dinucleotide (-A-), heptyl alpha-d-mannopyranoside (-B-), and tetrazole-based antifungal drug candidate VT1161 (VT1), respectively.

The mechanism of antifungal activities of the three major constituents in *F. communis* extracts was examined by docking to the crystal structure of sterol 14-alpha demethylase (CYP51) from *C. albicans* encoded in the PDB by 5TZ1.pdb. The binding energies were -5.88, -6.93, and -6.77 kcal/mol, corresponding to C7, C8, and C12, respectively (Fig. 10). The major compounds of the studied plant produced similar chemical interactions, with three hydrogen bonds created with Arg381, Tyr118, and His468 AAR, and more than one bond resulting in a Cys470 AAR in the A chain of the targeted protein. The docking validation protocol for these nine molecular simulations was successfully evaluated. The three major compounds of the *F. communis* plant were strongly docked to the active sites of each targeted protein, as confirmed with the assistance of the protein plus server (https://proteins.plus/)^51^. The active sites of the proteins were defined as co-crystallized ligands (or native ligands) (Fig. 11). Three amino acids, Ala11, Lys134, and Thr9, were active sites for the NADPH oxidase protein. Amino acids Asp47, Asp54, Asp140, Phe1, and Gln133 AARs were the active sites of the antibacterial protein. Arg381, Tyr118, and His468 are the active sites of antifungal proteins. Binding energies in Kcal/mol are primarily negative and do not exceed the − 5.000 kcal/mol threshold, demonstrating molecular stability^52^. These results are consistent with those of Jeddi et al.^53^, who demonstrated antioxidant and antibacterial effects toward the same targeted receptors, NADPH oxidase and FimH lectin proteins, for two major compounds of the essential oil extracted from the mill of *Lavandula angustifolia.* In that study, the major compounds in *F. communis* extracts were similarly docked to the active sites of each responsible protein, sharing broadly equivalent intermolecular interactions. The biological activity of antifungal molecules from natural sources toward lanosterol 14-alpha demethylase (CYP51) involves similar intermolecular interactions, including the active sites of the CYP51 protein from *C. albicans*^54^.

## Conclusion

In this study, we examined the effects of various solvents on extracts. The extracts demonstrated considerable antioxidant activity across various antioxidant methods, and exhibited antimicrobial activity against bacteria and yeasts. The extract contained moderate amounts of flavonoids and polyphenols. The bioactive substances identified in the non-volatile section of *F. communis* via the HPLC–DAD study are most likely responsible for these effects. Information on ligand–protein interactions involving antioxidant and antimicrobial proteins has provided valuable insights into binding affinities and interactions. These findings support further exploration via in vivo and in situ experiments, as well as the investigation of other biological activities of this plant.

## Data Availability

All data generated or analyzed during this study are included in this published article.
