# Author Correction: Comprehensive analysis of different solvent extracts of *Ferula communis* L. fruit reveals phenolic compounds and their biological properties via in vitro and in silico assays

**DOI:** 10.1038/s41598-024-63023-w

**Published:** 2024-05-31

**Authors:** Ghizlane Nouioura, Mohamed El fadili, Azeddin El Barnossi, El Hassania Loukili, Hassan Laaroussi, Mohammed Bouhrim, John P. Giesy, Mourad A. M. Aboul-Soud, Yazeed A. Al-Sheikh, Badiaa Lyoussi, El houssine Derwich

**Affiliations:** 1 Laboratory of Natural Substances, Pharmacology, Environment, Modeling, Health and Quality of Life (SNAMOPEQ), Faculty of Sciences Dhar El Mehraz, Sidi Mohamed Ben Abdellah University, 30 000 Fez, Morocco; 2 LIMAS Laboratory, Faculty of Sciences Dhar El Mehraz, Sidi Mohamed Ben Abdellah University, 30 000 Fez, Morocco; 3 Biological Engineering Laboratory, Faculty of Sciences and Techniques, Sultan Moulay Slimane University, Beni Mellal, Morocco; 4 Laboratory of Biotechnology, Environment, Agri-Food and Health, Faculty of Sciences Dhar El Mahraz, Sidi Mohammed Ben Abdellah University, 30050 Fez, Morocco; 5 Euromed Research Center, Euromed Polytechnic School, Euromed University of Fes, 30 000 Fez, Morocco; 6 Laboratory of Biological Engineering, Team of Functional and Pathological Biology, University Sultan Moulay Slimane, 23000 Beni Mellal, Morocco; 7 Department of Veterinary Biomedical Sciences and Toxicology Centre, University of Saskatchewan, Saskatoon, SK S7N 5B3 Canada; 8 Department of Integrative Biology and Center for Integrative Toxicology, Michigan State University, East Lansing, MI 48895 USA; 9 Department of Environmental Science, Baylor University, Waco, TX 76798 USA; 10 Department of Clinical Laboratory Sciences, College of Applied Medical Sciences, King Saud University, P.O. Box 10219, 11433 Riyadh, Saudi Arabia; 11 Unity of GC/MS and GC, City of Innovation, Sidi Mohamed Ben Abdellah University, Fez, Morocco

Correction to: *Scientific Reports* 10.1038/s41598-024-59087-3, published online 09 April 2024

The original version of this Article contained an error in the Acknowledgements section.

“The authors extend their appreciation to Researchers Supporting Project (No. RSPD2024R816), King Saud University, Riyadh, Saudi Arabia.”

now reads:

“The authors extend their appreciation to the Researchers Supporting Project (RSP 2024/520), King Saud University, Riyadh, Saudi Arabia, for their financial support. We also acknowledge the Prince Naif Health Research Center, Investigator Support Unit, for providing language editing services.”

In addition, the Funding section contained an error.

“This research was funded by the Researchers Supporting Project (No. RSPD2024R816), King Saud University, Riyadh, Saudi Arabia.”

now reads:

“This research was supported by the Researchers Supporting Project (RSP 2024/520), King Saud University, Riyadh, Saudi Arabia.”

The original Article has been corrected.

